# Adapting to Adversity: Effects of COVID-19 on Parenting in Chile

**DOI:** 10.3389/fpsyg.2022.868817

**Published:** 2022-07-05

**Authors:** J. Carola Pérez, Daniela Aldoney, Anastassia Vivanco-Carlevari, Soledad Coo, Eugenio J. Guzmán, Jaime R. Silva

**Affiliations:** ^1^Centro de Apego y Regulación Emocional, Universidad del Desarrollo, Santiago, Chile; ^2^Sociedad Desarrollo Emocional, Santiago, Chile; ^3^Department of Social Psychology, University of Amsterdam, Amsterdam, Netherlands; ^4^Clínica Alemana de Santiago, Santiago, Chile

**Keywords:** COVID-19, parenting, stress, depression, home chaos, parent–child interactions

## Abstract

The pandemic outbreak in March 2020 and its associated sanitary regulations and restrictions triggered an abrupt and significant change for society in general and for families’ organization in particular. In Chile, the Santiago Metropolitan District was under a strict lockdown that involved the closure of the entire educational system. From a systemic-family stress perspective, the impact of these changes might have consequences not only for each individual family member, but for the parental dynamic and, consequently, for children’s well-being. This paper presents the results of a follow-up study showing changes in self-reported parental depression and the perceived home organization of mothers and fathers assessed at three different moments: before the pandemic, at the initial outbreak, and after 1 month of strict lockdown. Relevant moderators were explored using linear mixed models to understand the within-subject changes in mothers’ and fathers’ self-reports across the different assessment times. Financial strain, personality traits of self-criticism and dependency, previous parent–child quality interaction, recent major stressful events, and number of children are highlighted as relevant factors that moderate changes in home chaos and parental mental health perception. Significant risks and protective factors are described for fathers and mothers. The use of pre-pandemic measures as baseline levels enabled the identification of personal and family characteristics that were related to better outcomes. The results help increase our understanding of the sanitary regulations’ impacts on the family system and identify vulnerability indicators that should be considered.

## Introduction

The current COVID-19 pandemic poses an acute threat to the well-being of children and families due to challenges related to a range of disruptions to daily functioning, such as financial insecurity, isolation, and confinement-related stress. Prime et al. (2020) illustrate the multitude of ways in which the well-being of children and families may be at risk during the COVID-19 pandemic through its effect on caregivers’ well-being. Diverse studies have already reported negative consequences of the pandemic and its associated sanitary restrictions on mental health and family functioning, such as increased anxiety, depression, and sleep problems (e.g., Brown et al., 2020; Huang and Zhao, 2020; Statistics Canada, 2020).

Moreover, lockdowns and school closures have disturbed family interactions by transforming the home into a workspace and school for many families, which challenges the organization of daily life. For example, Segre et al. (2021) found that children had difficulties adapting to different routines that resulted from new habits as products of the COVID-19 lockdowns and school closures. In this regard, lockdowns may result in more people remaining within the home for longer periods of time and may trigger changes to work schedules, which may lead to increased stress levels by imposing new demands on parents. These aspects together with the uncertainty about the future have had a profound impact on psychosocial outcomes, making research in this area a top priority (Holmes et al., 2020).

However, the impact of the pandemic and resultant adaptations in daily family life and family relationships remains largely unknown (Prime et al., 2020). Most literature does not include pre- and post-COVID-19 family data and focuses solely on mental health symptoms. Therefore, in this study we compare family functioning pre- and post-COVID-19 pandemic to examine the effect of this crisis on parents and family life. In addition, we examine how pre-existing parent and home-related variables were associated with such changes. Although potentially impactful for all individuals, preexisting individual and/or family characteristics (e.g., personality traits, poverty, and mental health) may function as risk factors by increasing the susceptibility to negative pandemic-related outcomes.

### Theoretical Background

The family stress model explains the process by which the experience of severe economic pressure undermines parents’ mental health, parenting, and subsequent child adjustment (Conger and Conger, 2002). Theoretically, the model suggests that emotional distress associated with severe economic pressure is the mechanism by which risk for poor parenting and child maladjustment increases (Conger and Conger, 2002). This model has been expanded to examine how other stressful events – besides economic hardship – may have an impact on family life. When stressors become frequent or if parents or families lack the resources to overcome them, individuals and subsystems within the family may present maladjustment, including physical, emotional, and mental health symptoms.

We conceptualize the COVID-19 pandemic as a stressful event with the potential to produce change in different family subsystems (Patterson, 1988). The pandemic-related negative impacts may be particularly significant for parents and their young children because proximal processes in the home environment during the early years of life are an important predictor of a child’s social and cognitive development (Bronfenbrenner and Evans, 2000). In addition, parenting young children is already a demanding task as caregivers are responsible not only for their children’s survival, but also for their children’s general well-being (Bornstein, 2014).

### Pandemic and Family Life

International research efforts show that the COVID-19 pandemic has generated extremely stressful conditions for individuals and families, resulting in a higher risk of developing mental health problems during COVID-19 outbreaks (Chaix et al., 2020; Wang et al., 2020). In a nationally representative Irish sample surveyed during the first week of government-imposed quarantine, 22.77% of people screened positive for depression, 20% screened positive for generalized anxiety, and 27.67% screened positive for both depression and anxiety (Hyland et al., 2020). A study of people from 190 Chinese cities found moderate-to-severe levels of stress, anxiety, and depression during the initial outbreak that persisted 4 weeks later (Wang et al., 2020). More recently, a meta-analysis including 16 studies that assessed 78,225 participants reported an average depression prevalence of 34.31%. A sub-group analysis showed that depression was higher in China (36.32%) than in other countries (28.3%) (Necho et al., 2021).

The COVID-19 pandemic has also impacted levels of home chaos within the family context. The home environment refers to the setting in which family interactions take place, shaping individual behavior and development (Bronfenbrenner and Crouter, 1983). For the early years of life, the home environment is the principal microsystem in which a child’s social and cognitive development takes place (Bronfenbrenner and Evans, 2000). Higher levels of home chaos (i.e., disorganization and instability) pose a significant risk for children’s adjustment, given their dependence on positive family processes for a host of developmental outcomes. Some of the COVID-19-related restrictions (e.g., school closure, social distancing, lockdowns, etc.) may lead to increased home chaos by imposing new demands on parents, especially those of younger children who need monitoring and assistance to successfully engage in distance learning.

Home chaos is connected to a variety of adverse consequences for parents and young children. Parents may experience the environment as more unpredictable and out of their hands, creating a feeling of diminished control. Perceived uncontrollability appears to make parents more vulnerable to a reduced sense of parental efficacy and well-being (Tucker et al., 2017). Also, continued exposure to noise and crowding may increase physical fatigue, which in turn makes parents less cooperative and more aggressive (Wachs, 1992). Children living in chaotic home environments present poorer cognitive competence, less adequate language development, greater likelihood of a more difficult temperament, reduced mastery and motivation, and an increased risk of accidental injury (Wachs, 2000).

Home chaos during the COVID-19 pandemic-related lockdowns has been associated with increases in maternal-child conflict, paternal-child conflict (Cassinat et al., 2021), higher levels of parenting stress in parents of children aged 2 to 14 years (Spinelli et al., 2020), and increased parental distress and household chaos among low-income families (Johnson et al., 2021). These recent studies highlight the relevance of understanding family interactions impacted by the pandemic restrictions as a specific context. Importantly, individual and context-specific characteristics should also be considered to understand which of them constitute risk factors for mental health problems and negative family interactions.

### Individual Differences: Risk or Protective Factors?

Individuals within the population vary greatly in their vulnerability to stressful life events (Sapolsky, 2015). A predisposition to maladjustment may arise in a variety of ways and at different stages of the life cycle. To develop targeted preventions and interventions aimed at those most vulnerable, we need to examine individual and family characteristics that influence well-being during the pandemic.

Research reveals several factors predicting differences in individual and family responses to stressful contexts, such as the COVID-19 pandemic. Some of these are low socioeconomic status, maladaptive personality traits, negative affect, and being female (Cao et al., 2020; Flesia et al., 2020; Hyland et al., 2020; Mazza et al., 2020). For example, people with low income are more likely to face chaotic living conditions than are their middle- and upper-income counterparts. In addition, economic-related stress and limited resources make them less equipped to circumvent or overcome chaos-related challenges (Evans et al., 2010). Other factors such as number of children, number of lockdown days, and number of stressful events are demands that diminished a parent’s ability to deal with added stressors, such as those related to the ones experienced due to the COVID-19 pandemic (Kessler, 1997; Olhaberry and Farkas, 2012).

From a different perspective, personality characteristics are also suggested as influencing individual responses to stressful events. Specifically, some of these are associated with a high risk of developing symptoms of depression. Accordingly, Blatt’s theory of depressive personality style describes self-criticism and dependency as predisposing the development of these symptoms (Blatt, 2008). Self-critical thinking takes place when specific standards are not met. Individuals who present strong self-criticism traits often hold very high expectations and standards for themselves, and fear disapproval and loss of control, which render them vulnerable to experiencing feelings of failure, worthlessness, and guilt. Dependency is related to intense fears of abandonment and loss, a high need to feel loved, and a strong tendency to seek support and help from others (Luyten et al., 2007). Experimental studies have shown that variations in dependency and self-criticism correlate not only with different subjective responses to stress, but also with differential sensitivities in the neurohormonal response to stress (i.e., cortisol response to interpersonal stress) (Silva et al., 2017). These characteristics may interfere with the capacity to effectively organize a complex home environment and may increase relationship stress, which in turn are negatively related to child development (Tang et al., 2016).

Given the systemic aspects of family relations, the quality of parent–child relationships may also play a role as a protective or risk factor for the negative impacts of the COVID-19 pandemic on families (Doty et al., 2017). Assessing parenting behaviors early in development is important because diverse studies suggest that parenting at an earlier time point robustly predicts parenting at a later time point (Dallaire and Weinraub, 2005). The relationship between parenting, depression and home chaos has been established in the literature. In general, parents who display positive (i.e., warmth, sensitivity or responsiveness) rather than negative (negative control, negative affect) parenting practices create a better context for development (Vandell et al., 2010). A meta-analysis concluded that depression was associated with negative maternal behavior (Lovejoy et al., 2000). Other studies have found a relationship between negative parenting and higher levels of home chaos (Corapci and Wachs, 2002). Given that depression, household chaos, and parenting quality are in part dependent upon or manifested in the parents themselves, it is highly likely that the relation between parenting and home chaos and depression is of a transactional nature (Garrett-Peters et al., 2019).

Family characteristics may also place some parents at greater risk for negative consequences in their parenting practices and well-being during the pandemic. Compared to middle-class families, low-income families tend to experience more severe consequences associated with insufficient financial resources, augmenting the negative effect of financial stress on caregivers’ mental health (Ponnet, 2014) with consequences for children’s adjustment (Neppl et al., 2016). These characteristics may be exacerbated due to COVID-19 pandemic-related disruptions.

In order to develop targeted preventions and interventions aimed at those most vulnerable, we need to examine individual and family difference variables that influence well-being during the pandemic. Furthermore, despite similarities across different global regions, it is important to consider the specific local characteristics of sanitary measures, socioeconomic and political contexts, and populations’ reactions.

### The COVID-19 Pandemic in Chile

The first cases of COVID-19 were detected in Chile in March 2020 in a context of social, political, and economic crisis associated with an earlier social protest outburst that took place in October 2019. This movement demanded equity and justice (Caqueo-Urízar et al., 2020). To prevent the spread of the virus, Chilean authorities imposed drastic measures in mid-March, including restrictive lockdowns, a national curfew, business closings and the complete shutdown of the educational system (i.e., daycares, schools, and universities) (Fischman and Irarrazaval, 2020). By the beginning of May, almost the entire Metropolitan District was placed in strict lockdown. These measures lasted between 3 and 4 months for the different areas of Santiago (i.e., the capital city) and for urban areas in the rest of the country, with significant consequences in several dimensions, including mental health and socioeconomic aspects. Consistent with international data, feelings of fear, anxiety, and sleep problems significantly increased in the general population (Dagnino et al., 2020).

From a socioeconomic perspective, the interruption of the normal functioning of diverse economic activities due to the pandemic-related restrictions led to job losses and household income reductions, especially for families with children and adolescents, where six out of ten households (59.4%) faced a decrease in income compared to their pre-pandemic situation (UNICEF et al., 2021). This was particularly significant for low socioeconomic groups and for female-headed households; 57.8% presented a decrease in their income compared to 50.8% of male-headed households, thus worsening the existent patterns of inequality in the country (UNICEF et al., 2021). Furthermore, the unemployment rate stood at 13.1% from May to July, its highest of the past 16 years (INE, 2020). These specific contextual factors should be considered in order to understand the specific state of affairs in Chile and for the families that participated in this study.

In view of the complex relationship between mental health, parenting, and home organization, and the dramatic impact of the COVID-19 pandemic in Chile, this exploratory study examined the associations between a broad set of risk factors with parental depression and home chaos in the Chilean context during the health crisis. Specifically, we examined (1) how parental depressive symptoms and home chaos changed from before the pandemic to the initial lockdown; (2) if different parental characteristics (i.e., parental self-criticism, dependency, and positive/negative control in interactions with children) and family variables (i.e., economic strain and number of children) were associated with parental symptoms of depression and family chaos at the onset of the pandemic; (3) interaction effects to test if personal and family variables moderated the changes in depression symptoms and home chaos from before the pandemic to the initial lockdown.

We hypothesized an increase in depression and home chaos from the pre-pandemic period (T1) to the onset of lockdown (T2) and after 40 days of lockdown (T3). We also expected that stressful circumstances (i.e., longer periods of lockdown, financial difficulties) and personality traits (i.e., self-criticism and dependency) negatively contribute to parents’ ability to adapt to the COVID-19 pandemic-related challenges, impairing their emotional well-being and family functioning.

## Materials and Methods

### Participants

This study used a repeated-measures design to follow up on a sub-sample of 68 families who were involved in a larger study from the period before the COVID-19 pandemic (T1) to the onset of the lockdown restrictions (T2) and 40 days later (T3). Inclusion criteria were: (1) being an adult (i.e., 18 years of age or older), (2) cohabiting parents, (3) with a child aged 3 to 4 years at T1. Exclusion criteria for parents referred to diagnosis of an intellectual impairment, neurodevelopmental or severe psychiatric disorder (i.e., schizophrenia, bipolar disorder, major depressive disorder).

The T1 sample consisted of 120 low- to middle-income Chilean families. All children were living with both parents. Children’s mean age at the first assessment was 35.78 months (SD = 3.77, range = 29–46), 47% were boys, and 51.3% were enrolled in childcare. The mothers’ and fathers’ mean age was 31.15 years (SD = 6.10) and 33.9 years (SD = 7.09), respectively. Most of the mothers (87%) and fathers (86%) reported having earned a high school diploma or beyond.

Only 77 families were reachable at T2 (64% of T1 sample) and invited to be part of this follow-up (T2 and T3) with 68 (88%) of these families agreeing to participate. At T2, most mothers (80.9%) reported living with their partner (i.e., the child’s other parent) and 98.5% reported living with their child/children all of the time. The remaining 1.5% of mothers reported living with their child/children only some days per week. With regard to the number of children per household, 26.5% of the mothers reported having only one child, 33.8% had two, 35.3% had three and 4.4% had four or more. The mothers’ educational levels were distributed as follows: 8.8% did not finish secondary school, 26.5% completed secondary school, 36.8% did not finish higher education, 25% completed college or higher education. Taking into account paternal and maternal reports of combined monthly family income, 7.8% of our sample reported earning less than USD 250, 12.5% earned between USD 250 and 380, 29.7% earned between USD 380 and 640, 35.9% between USD 640 and 1,300 and 14.1% reported a monthly family income higher than USD 1,300. Maternal and paternal reports showed some small differences (see Table 1).

**TABLE 1:** Sample demographic characteristics at T2.

	Mother	Father
N° of children	*n* = 68	*n* = 65
1	26.5%	26.2%
2	33.8%	36.9%
3	35.3%	32.3%
4	2.9%	3.1%
5	1.5%	1.5%
Parent living with child	*n* = 68	*n* = 63
Yes	98.5%	82.5%
Some days a week	1.5%	7.9%
No	–	9.5%
Parent living with partner	*n* = 68	*n* = 63
Yes	80.9%	82.5%
No	19.1%	17.5%
Educational information	*n* = 68	*n* = 65
Incomplete primary school	1.5%	–
Complete primary school	2.9%	3.1%
Incomplete secondary school	4.4%	4.7%
Complete secondary school	26.5%	31.3%
Incomplete higher education	36.8%	26.6%
Complete higher education	25%	9.4%
Other	2.9%	25%
Household income	*n* = 64	*n* = 58
Less than $250 USD	7.8%	–
Between $250 – $380 USD	12.5%	13.8%
Between $380 – $640 USD	29.7%	32.8%
Between $640 – $1300 USD	35.9%	36.2%
More than $1300 USD	14.1%	17.2%

In Chile, 88% of the population minimally holds a higher education diploma and the mean year of completed education is 11.05. Families in our study are considered low- or middle-income if they have an average monthly household income between USD 734 and 1,468 (Ministerio de Desarrollo Social y Familia [MDSF], 2017). Currently, mean income in Chile stands at USD 796 per month and minimum wage is set at USD 415 per month.

### Procedures

For T1 (2018), we recruited participants through wall posters inviting mothers and fathers of children between the ages of 2.5 and 3.5 years to participate in a study on play, learning, and development (see Supplementary Figure 1) or by approaching them in the waiting rooms of four primary health care centers in the southern part of Chile’s capital, Santiago. We contacted interested families by phone for further explanation of the project and scheduled appointments for the assessments that were conducted at the health care centers. Mothers and fathers completed questionnaires on paper during the assessments. Each family received an educational toy for their child, and children were given a sticker for participating. For T2 (April 2020) and T3 (40 days after T2) families were contacted via phone to invite them to participate in the follow-up study. Families who agreed to participate where informed over the phone about the study characteristics and had the opportunity to ask questions about the procedure. Families received the study information sheet and informed consent, which stated the study’s general purpose, the instrument’s length, and the contact details of the principal investigator. Those who agreed to participate completed a set of online questionnaires when mandatory quarantine was implemented in Santiago (T2) and again 40 days later (T3). Each questionnaire was designed to fit on the screen, resulting in five screen views of questions for participants to read and complete. All participants received USD 7 upon completion. The study procedures were approved by the University Institutional Review Board. For more information on the recruitment process and survey characteristics, see Supplementary Table 1.

### Instruments

#### Depression

At T1, T2, and T3, parents completed the Spanish version of the Depression Scale of the Center for Epidemiological Studies (CES-D; Radloff, 1977; Eaton et al., 2004). The CES-D is a ten-item, self-report questionnaire that assesses depressive symptoms over the past week. The total score ranges from 0 to 30 and higher scores show greater symptom severity. Scores higher than 10 indicate risk of presenting a depressive disorder. The Cronbach’s α of the scale across the three assessment times ranged from 0.78 to 0.89 for maternal reports, from 0.76 to 0.85 for paternal reports, and from 0.80 to 0.87 for maternal and paternal reports combined.

#### Home Chaos

At T1, T2, and T3, mothers and fathers completed the CHAOS scale (Confusion, Hubbub, and Order Scale; Matheny et al., 1995) that evaluates routines and home organization. This questionnaire was translated into Spanish and is used with various Spanish-speaking populations (Haack et al., 2011). The total score range for this scale is 15 to 75 with higher scores reflecting a greater perception of chaos and disorganization in the home. Cronbach’s α of the scale across the three assessment times ranged from 0.80 to 0.88 for maternal reports, from 0.75 to 0.85 for paternal reports, and from 0.78 to 0.87 for the combined maternal and paternal reports.

#### Sociodemographic Information

At T1 and T2, mothers and fathers reported their age, number of children, level of education, and monthly family household income. Participants also reported on a number of stressful events that occurred between T1 and T2 (10 event options including accidents, unemployment, family member’s death, divorce, and others).

#### Quality of Parent–Child Interactions

At T1 the mother–child and father–child free-play interactions were coded using the Parent–Child Interaction System (PARCHISY; Deater-Deckard and Dodge, 1997). For the purpose of this study, only positive and negative control coding were used; positive control (i.e., use of praise, explanations and open questions) and negative control (i.e., redirection of the child’s behavior, paying little or no attention to the child’s interests, negative control) were coded on a seven-point Likert scale ranging from very low (1) to very high (7). All father–child and mother–child interactions were coded independently by two researchers (trained by the PI) with good inter-rater reliability for 25% of the entire sample (Cronbach’s α = 0.93).

#### Personality Traits

The Depressive Experiences Questionnaire (DEQ; Blatt et al., 1976) from the polarities of experience model (Blatt, 1974) was completed by fathers and mothers at the final assessment (T3). This 66-item instrument has been widely used in personality and character styles research (Zuroff et al., 1990). Dependency and self-criticism personality dimensions are obtained from this tool following the scoring method instructed in the original description of the scale (Blatt et al., 1976). The scores ranged from −3.5 to +3.5 points (standardized score). The estimation of the subscale scores considers all the items, but they are weighted differently in the subscales. Therefore, the reliability of the scale as a whole was calculated using Cronbach’s alpha (value 0.87).

#### COVID-19 Experience

At T2 and T3 parents reported the socio-economic consequences of the COVID-19 crisis for the item “*My economic situation has been affected by coronavirus*” on a seven-point Likert scale (1 = no consequences – 7 = severe consequences). The number of lockdown days was also reported by a single item; range between no confinement (score zero), 1 week of confinement (score 1) sequentially until 8 weeks of confinement and nine or more weeks of confinement (score 9).

### Analysis

We conducted a descriptive analysis of the data and estimated Pearson correlations between variables. Also, mothers’ and fathers’ ratings were compared with an dependent-samples *t*-test. For each one of the dependent variables (i.e., depressive symptoms and CHAOS), a two-piece discontinuous model of change was modeled with a linear mixed model (LMM). The analyses were conducted with IBM-SPSS v.23 (REML estimation method) using a model-building approach (Hoffman, 2015). Based on theoretical considerations and our descriptive results, we selected the following variables to test in the moderation analysis: negative control, self-criticism and economic strain.

From a theoretical perspective, the variance of dependent variables could be included in a three-level model; three time points (Level 1) were nested within persons (Level 2) and then nested within families (Level 3). It is reasonable to propose that mothers and fathers belonging to the same family have some degree of data non-independence based on their kinship linkage or their daily interactions (Kenny et al., 2006). Nevertheless, this assumption must be evaluated empirically.

In the case of parental depressive symptoms, using the ML-2ΔLL test (Hoffman, 2015) a single-level model (independent data) was compared to a two-level model (time points nested within persons), and the latter was compared to a three-level model (time points nested within persons, and both nested within families), showing that a two-level model was more adequate than a single model, ML-2ΔLL test = 46.3 (1 df.), *p* < 0.001. Nevertheless, the three-level model fit did not improve with respect to the previous one, ML-2ΔLL test = 1.0 (1 df.), *p* = 0.307, indicating that there was no between-family intercept variation. Thus, a two-level model composed of time points nested within persons was created.

First, the null model was estimated to account for the intra-class correlation (ICC). We then described the change over time (step 1). In this step, the two-pieces discontinuous model fixed the intercept at the beginning of lockdown (indicating the value of parental depressive symptoms at T2). The first slope accounted for the pre-COVID-19 change (T1 to T2, coded as −1), and the second slope accounted for change during the lockdown period (T2 to T3, coded as +1). Based on data variability, a random intercept and pre-COVID-19 slope were modeled. Their covariance was also estimated.

In the second step, several individual (i.e., mother, child gender, parent age, stressful events and dimensions of polarities of the experience) and contextual predictors (i.e., economic strain and lockdown days due to COVID-19, number of children, positive and negative parental control) were included to explain the intercept. The predictors were identified following preliminary analyses and according to their theoretical relevance. In step three, some specific interaction terms were included to account for differences in pre-COVID-19 changes in parental depressive symptoms. This model incorporated all significant step 2 predictors. All predictors were centered with respect to their mean (except the mother variable).

A similar procedure was followed for the chaos variable. Before establishing the models, we determined which levels should be included. This analysis shows that the two-level model was more adequate than a single model, ML-2ΔLL test = 52.1 (1 df.), *p* < 0.001, and that the three-level model fit did improve with respect to the previous one, ML-2ΔLL test = 11.6 (1 df.), *p* < 0.001. Consequently, in this case, a three-level model composed of three time points nested within persons and in turn nested within families was devised. It was necessary to model level −1 as a diagonal matrix to the Hessian matrix for convergence.

In the first step, the two-piece discontinuous model fixed the intercept at the beginning of lockdown, and one of the slopes accounted for the pre-COVID-19 change and the other one accounted for change during the lockdown period. Based on data variability, a random intercept and pre-COVID-19 slope were modeled at level two (person) and level three (family). Finally, their respective covariance was estimated only at level three.

In the second step, in addition to individual and contextual predictors, we included “family variables.” These variables were composed as a mean of the mother’s and father’s values in each respective family. This procedure considered all predictors that could capture differences within families (e.g., the mothers report more stressful events than their partners) and between families (e.g., parents of one family report more stressful events than the others). This procedure was done based on Hoffman (2015), who recommends it in order to prevent conflating the variance of person-level (mother or father report) with the variance associated with family level. Accordingly, most predictors (except mother, child’s sex and number of children variables, which are fixed within families) were modeled by two parameters; one that captures within-family variance and another that captures between-family variance. Both parameters were group-mean centered.

Lastly, interaction terms were included to account for differences in pre-COVID-19 changes in chaos (step three). This model incorporated all significant step-two predictors and hypothesized interaction terms (parameters to model within and between variance were modeled together).

Additionally, we used a Monte Carlo simulation to estimate the observed power of fixed effects. This procedure assumes that the estimated parameter values of the sample are the true population values for these parameters. Based on this assumption, 1,000 replication samples were generated from this population model using the original sample size and their time points per subject to determine the number of samples in which certain parameters are statistically significant (Bolger and Laurenceau, 2013). This percentage of samples capture the statistical power. The *post-hoc* power estimated for each fixed parameter included in the model was reported in the corresponding table.

## Results

### Descriptive Results

The descriptive characteristics of the participants are in Table 2. Our results show that at the three assessment times, mothers reported higher levels of depression than their male partners [T1: *t*(64) = 2.46, *p* = 0.017; T2: *t*(62) = 4.30, *p* < 0.001; T3: *t*(64) = 2.46, *p* = 0.017]. We found a similar pattern for maternal reports of home chaos, which were higher than paternal reports at T1 and T2 [T1: *t*(64) = 2.08, *p* = 0.041; T2: *t*(61) = 2.44, *p* = 0.018]. No differences between mothers and fathers were found in this variable at T3. Mothers also reported being confined for longer periods of time than fathers [*t*(58) = 2.826, *p* = 0.006].

**TABLE 2:** Descriptive data of mother’s and father’s characteristics and context.

	Mother report			Father report		
		
	*M*	*SD*	*n*	*M*	*SD*	*n*
1. Child sex (1 = female)	0.52	0.50	65	0.52	0.50	65
2. Adult’s age	31.44	5.82	64	33.49	6.82	65
3. Lockdown duration	2.96	1.28	67	2.30	1.38	60
4. Economic strain	4.62	2.12	68	4.61	2.11	62
5. Number of children	2.19	0.92	68	2.17	0.91	65
6. Stressful events	1.88	1.31	68	1.76	1.01	63
7. Negative control	2.02	1.30	63	1.70	0.81	64
8. Positive control	4.57	1.09	63	4.47	1.31	64
9. Dependency	−0.95	0.70	62	−1.06	0.59	60
10. Self-criticism	−0.87	0.85	62	−0.99	0.86	60
11. Depressive symptoms time 1	8.62	5.33	65	6.66	5.05	65
12. Depressive symptoms time 2	12.35	5.11	68	8.84	5.24	63
13. Depressive symptoms time 3	12.73	6.48	63	9.85	5.40	59
14. Chaos time 1	37.95	8.73	65	35.90	6.68	63
15. Chaos time 2	56.15	7.40	66	53.76	6.85	63
16. Chaos time 3	54.34	7.82	62	52.05	6.87	60

We found no significant differences between mothers and fathers in terms of the number of stressful events experienced between the first assessment (T1) and the beginning of lockdown (T2) [*t*(62) = 0.30, *p* = 0.766], and the perception of change in financial security due to the COVID-19 pandemic [*t*(61) = 0.07, *p* = 0.945]. Similarly, mothers and fathers provided similar reports on dependency [*t*(55) = 1.38, *p* = 0.173] and self-criticism [*t*(55) = 1.51, *p* = 0.136] and the use of positive [*t*(61) = 0.61, *p* = 0.545] and negative control parenting [*t*(61) = 1.56, *p* = 0.123].

Most maternal and paternal reports on the study variables were not significantly correlated (see Table 3), yet the maternal perception of increased financial insecurity due to the COVID-19 pandemic was significantly associated with higher symptoms of paternal depression at T3. Also, larger numbers of children reported by mothers was positively associated with the level of chaos perceived by fathers at T1, although both parents reported having a similar number of children (*r* = 0.95, *p* < 0.001). In contrast, the larger number of children reported by fathers was related to higher maternal perception of home chaos during the three assessment times. With regard to the number of stressful events, maternal reports were positively associated with symptoms of maternal depression at T2 and with paternal reports of home chaos at T3. Similarly, paternal reports of more frequent stressful events were positively associated with maternal symptoms of depression and perception of home chaos at T3.

**TABLE 3:** Correlations between study variables.

Mother report	Father report															
	
	1	2	3	4	5	6	7	8	9	10	11	12	13	14	15	16
1. Child sex (1 = female)	1***	−0.30*	–0.15	−0.30*	–0.16	0.16	–0.04	0.00	0.04	–0.14	0.05	–0.03	–0.08	0.01	–0.06	0.11
2. Adult’s age	–0.17	0.82***	0.14	–0.04	0.61***	–0.02	0.04	0.13	0.20	–0.22	0.07	–0.19	–0.07	0.07	0.21	–0.03
3. Number of lockdown days	–0.13	0.16	0.09	–0.13	–0.14	–0.08	−0.29*	0.07	–0.03	–0.07	−0.28*	–0.13	–0.17	−0.28*	0.06	–0.24
4. Economic strain	−0.32**	0.12	–0.14	0.63***	0.17	0.14	–0.11	–0.19	0.01	0.26*	–0.13	0.14	0.29*	–0.21	0.16	0.20
5. Number of children	–0.16	0.46***	–0.04	0.09	0.95***	0.18	0.01	0.07	0.13	–0.15	0.19	–0.16	–0.16	0.26*	0.23	0.10
6. Stressful situations	–0.15	–0.23	–0.08	0.22	–0.19	0.41***	–0.09	0.06	–0.01	0.35**	0.14	0.27*	0.23	0.09	0.16	0.31*
7. Negative control	–0.21	0.05	0.12	0.18	–0.18	–0.12	0.20	–0.22	0.02	–0.05	–0.14	–0.09	0.11	–0.01	0.02	–0.08
8. Positive control	0.35**	0.03	0.90	–0.11	0.23	0.35**	–0.10	0.28*	–0.00	0.04	0.17	–0.09	–0.07	0.07	0.02	0.08
9. Dependency	–0.08	–0.05	–0.15	–0.04	–0.09	0.01	0.02	0.02	0.09	–0.04	–0.07	0.11	–0.06	–0.06	–0.05	–0.06
10. Self-criticism	–0.18	−0.27*	–0.09	–0.09	0.13	0.12	–0.07	–0.06	–0.08	0.24	–0.12	0.21	0.21	–0.07	–0.06	0.09
11. Depressive S. T1	–0.17	−0.30*	–0.05	–0.01	0.09	0.14	–0.08	0.19	–0.05	0.12	0.24	0.07	0.08	0.06	0.03	0.01
12. Depressive S. T2	–0.19	–0.03	–0.02	0.03	0.08	0.14	–0.06	–0.01	0.15	0.14	0.04	0.22	0.08	–0.08	0.14	0.10
13. Depressive S. T3	–0.22	0.01	–0.15	–0.05	0.18	0.23*	–0.19	0.09	0.04	0.13	0.04	0.29*	0.25	–0.07	0.11	0.22
14. Chaos T1	–0.21	0.04	–0.11	0.04	0.39***	0.22	0.13	0.01	0.07	0.10	0.16	0.00	0.12	0.39***	0.37**	0.15
15. Chaos T2	0.133	0.18	–0.19	–0.18	0.37***	0.22	−14	0.12	0.03	–0.11	0.02	0.02	–0.10	0.07	0.42***	0.29*
16. Chaos T3	–0.07	0.18	–0.21	–0.02	0.49***	0.34**	–0.21	0.16	–0.01	0.07	–0.07	0.07	0.11	–0.06	0.39**	0.42***

S, Symptoms. Correlations were estimated using pairwise deletion. *p < 0.05; **p < 0.01; ***p < 0.001.

Interestingly, maternal and paternal reports of depression were not significantly correlated (0.21 > *r* > 0.26; all *p*-values were non-significant). However, their reports of home chaos were positively associated, despite the differences in the average perception of home chaos between mothers and fathers (0.38 > *r* > 0.43, all *p* < 0.001).

### Depressive Symptoms

Of the total variation of depression symptoms over time, 38% (ICC = 0.38) corresponds to between-person differences and the remaining amount corresponds to within-person differences. Parents reported a significant increase in depressive symptoms from T1 (*M* = 7.62) to T2 (*M* = 10.69), but not compared to T3 (*M* = 11.40). At the beginning of the lockdown (intercept), mothers reported higher levels of depressive symptoms (β = 2.03, *p* < 0.01) than fathers. Both the dependency and self-criticism personality factors were positively related to parental depressive symptoms at the beginning of the lockdown due to the COVID-19 pandemic (T2). No other variables were related to parental depressive symptoms during this assessment time (see Table 4 for detailed parameters).

**TABLE 4:** Model of individual and contextual variables on parental depression.

Fixed effects	Step 1		Step 2		Step 3	
			
	Estimate	*Post-hoc* power	Estimate	*Post-hoc* power	Estimate	*Post-hoc* power
Intercept*^a^*	10.69***	0.99	9.53***	0.18	9.61***	0.99
Slope pre-COVID	3.07***	0.86	3.11***	0.99	3.15***	0.99
Slope during lockdown	0.73	0.38	0.80	0.47	0.74	0.44
***Control variables***						
Mother*^b^*	–	–	2.03**	0.90	1.92**	0.87
Child sex (1 = female)	–	–	−0.43	0.07	–	–
Adult’s age	–	–	−0.07	0.30	–	–
Stressful situations	–	–	0.33	0.62	–	–
***Contextual variables***						
Economic strain	–	–	0.14	0.26	0.35	0.77
Number of children	–	–	0.58	0.97	–	–
Lockdown duration	–	–	0.02	0.07	–	–
Parental negative control	–	–	0.09	0.15	−0.13	0.16
Parental positive control	–	–	0.17	0.30	–	–
***Individual characteristics***						
Dependency	–	–	1.25*	0.99	1.43**	0.99
Self-criticism	–	–	2.39***	0.99	3.49***	0.99
***Interactions***						
Slope pre-COVID * Economic strain	–	–	–	–	0.54*	0.80
Slope pre-COVID * Self-criticism	–	–	–	–	1.75**	0.99
Slope pre-COVID * Parental negative control	–	–	–	–	−0.55	0.70
**Random effects**						
Random intercept variance (Level-2: Person)	22.60***		11.76***		10.48***	
Random slope: Pre-COVID Variance	18.42***		15.60***		12.20**	
Intercept slope: Pre-COVID covariance	12.18***		7.98**		6.04*	
Residual variance	11.03***		11.48***		11.43***	

*Level-2, N = 136; Level-1, N = 408.*

*^a^Intercept was modeled by slope pre-COVID = 0 and slope during lockdown = 0; and time variables slope pre-COVID = −1 and slope during lockdown = 1.*

*^b^All predictors are grand-mean centered, except by mother (1 = mother; 0 = father).*

*p < 0.05; **p < 0.01; *** p < 0.001.

**FIGURE 1:**
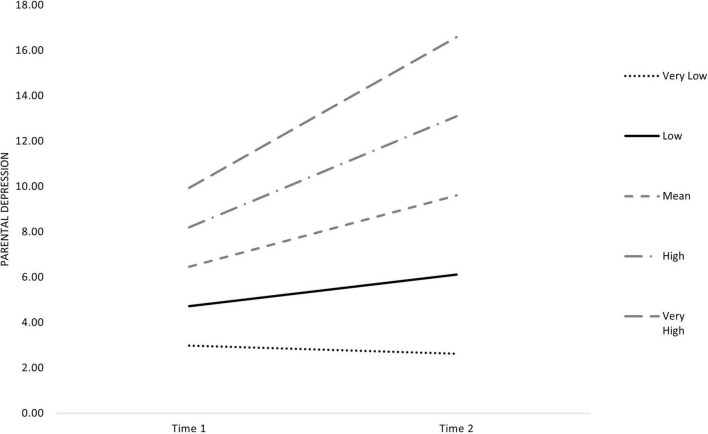
Changes in parental depression according to self-criticism level.

Focusing on the change in parental depressive symptoms, the interaction term indicated that parents with high scores in self-criticism showed a steeper slope increase in depressive symptoms from T1 to T2 (Figure 1).

**FIGURE 2:**
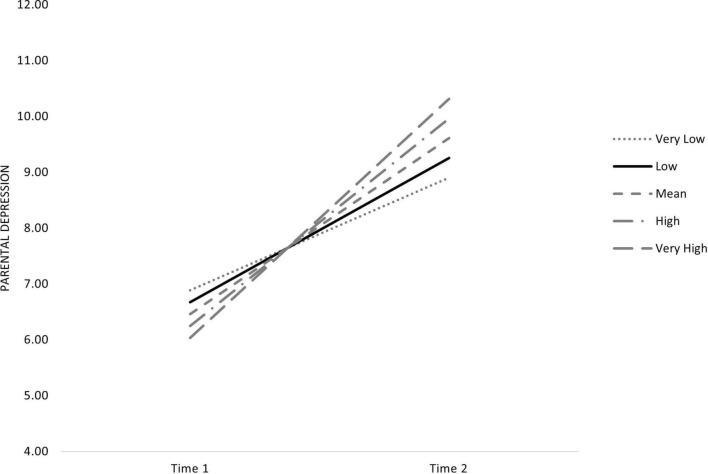
Changes in parental depression according to economic strain.

Finally, although the deterioration of the economic situation is not related to the level of depressive symptoms at the onset of lockdown (T2, β = 0.35, *p* = 0.192), the increase in parental depressive symptomatology between time 1 (2018) and that moment (T2) depends on the level of economic deterioration linked to the pandemic crisis. Therefore, a steeper increase in parental depressive symptoms occurred in families whose parents experienced greater deterioration in their economic situation (Figure 2).

### Home Chaos

The levels of home chaos showed a significant increase from T1 (*M* = 36.80) to T2 (*M* = 55.05) and a small but significant decrease from T2 to T3 *(M* = 53.33). At the beginning of the lockdown (intercept), bigger families (β = 2.81, *p* < 0.01) who had experienced more stressful situations (β = 1.69, *p* < 0.05) and where the parents had higher levels of self-criticism (β = 3.33, *p* < 0.001) reported higher levels of home chaos. No other variables were related to home chaos during this assessment time (see Table 5 for detailed parameters).

**TABLE 5:** Model of individual and contextual variables on chaos.

Fixed effects	Step 1		Step 2		Step 3	
			
	Estimate	*Post-hoc* power	Estimate	*Post-hoc* power	Estimate	*Post-hoc* power
Intercept*^a^*	55.05***	0.99	51.06***	0.99	51.36***	0.99
Slope pre-COVID	18.25***	0.99	18.06***	0.99	16.56***	0.99
Slope during lockdown	−1.72**	0.84	−1.54*	0.89	−1.52*	0.88
**Control**						
Mother*^b^*	–		1.78	0.53	–	
Child sex (1 = female)*^c^*	–		0.29	0.10	–	
Within-family: Parent age	–		0.05	0.09	–	
Between-family: Parents age	–		0.10	0.29	–	
Within-family: Stressful situations	–		−0.84	0.07	−0.99	0.06
Between-family: Stressful situations	–		1.69*	0.89	1.22*^T^*	0.69
**Contextual variables**						
Within-family: Economic strain	–		0.65	0.28	0.66	0.26
Between-family: Economic strain	–		−0.44	0.36	−0.18	0.13
Number of children*^c^*	–		2.81**	0.99	3.17***	0.99
Within-family: Lockdown duration	–		0.20	0.16	–	
Between-family: Lockdown days	–		−0.45	0.41	–	
Within-family: Parental negative control	–		0.07	0.06	0.14	0.07
Between-family: Parental negative control	–		0.56	0.20	−0.13	0.06
Within-family: Parental positive control			0.03	0.07		
Between-family: Parental positive control			−0.64	0.26		
**Individual characteristics**						
Within-family: Dependency	–		−0.68	0.13	–	
Between family: Dependency	–		−0.66	0.15	–	
Within-family: Self-criticism	–		3.33***	0.94	4.00***	0.97
Between-family: Self-criticism	–		1.52	0.55	1.84	0.57
**Interactions**						
Slope pre-COVID * Within-family Economic strain	–		-		−0.31	0.08
Slope pre-COVID * Between-family Economic strain	–		–		0.44	0.19
Slope pre-COVID * Within-family Self-criticism	–		–		1.68	0.16
Slope pre-COVID * Between-family Self-criticism	–		–		0.25	0.09
Slope pre-COVID * Within-family Parental negative control	–		–		−1.24	0.23
Slope pre-COVID * Between-family Parental negative control	–		–		−2.26*	0.56
**Random effects**						
Level-3 intercept variance (Level-3: Family)	17.82**		17.02**		16.39**	
Level-3 pre-COVID-slope	27.89***		28.73**		26.37**	
Level-3 intercept-slope pre-COVID covariance	11.81*		14.67*		13.18*	
Level-2 intercept variance (Level-2: Person)	16.94***		10.73*		10.42**	
Level-2 pre-COVID-slope	16.94***		10.73*		10.42**	
Residual variance time 1	4.77		16.21		15.11	
Residual variance time 2	21.33***		22.40***		21.12***	
Residual variance time 3	17.00***		15.48***		15.87***	

*Level-3, N = 68; Level-2, N = 136; Level-1, N = 408.*

*^a^Intercept was modeled by slope pre-COVID = 0 and slope during lockdown = 0; and time variables slope pre-COVID = −1 and slope during lockdown = 1.*

*^b^Mother (1 = mother; 0 = father).*

*^c^Grand mean centered. All other predictors are group mean centered.*

T < 0.06; *p < 0.05; **p < 0.01; ***p < 0.001.

**FIGURE 3:**
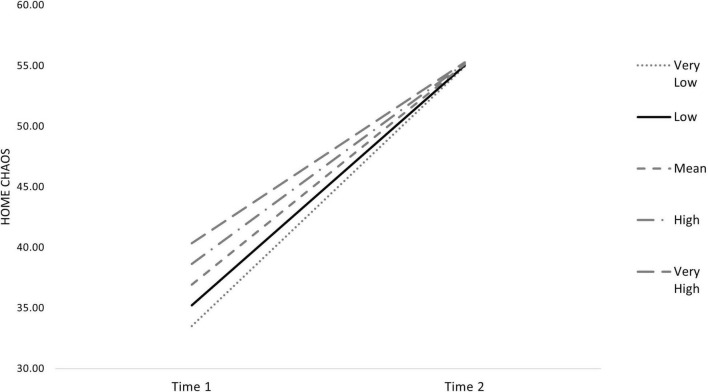
Changes in home chaos according to parental negative control.

Finally, only one of the interaction terms was significant, indicating that families with higher levels of negative control showed a less prominent change in home chaos from T1 to T2 (Figure 3).

## Discussion

This study examined associations between a broad set of risk factors with parental depression and home chaos in the context of the COVID-19 pandemic in a Chilean sample. The parents in our study reported an increase in symptoms of depression from the period before the onset of the pandemic to the start of the lockdown, these symptoms remained high later on during the pandemic. This is in line with previous studies, which have consistently reported a significant decrease in emotional well-being during the COVID-19 pandemic (Wang et al., 2020; World Health Organization, 2020). Our results show interesting associations between parental characteristics and lockdown-related aspects, which may explain the persistent increase in reports of depression.

Another relevant variable contributing to parental depression was financial hardship. In our study, both mothers and fathers who reported larger increases in financial hardship related to the pandemic also showed a larger increase in symptoms of depression from the period before the onset of the pandemic to the start of the lockdown. A recent cross-sectional study conducted in the United Kingdom found similar results, showing that greater financial hardship experienced during the pandemic was related to poorer mental health in adults (Smith et al., 2020). This is particularly relevant for our national context in which studies prior to the COVID-19 pandemic showed a high frequency of depressive disorders (Toledo et al., 2015) and substance abuse (González Suitt et al., 2019), which were aggravated by socioeconomic inequalities (Rotarou and Sakellariou, 2017).

Interestingly, high financial stress in our study as reported by mothers was moderately correlated with paternal depression. Considering the traditional role division that often exists in Chilean families where men have more responsibilities as family providers (Aguayo et al., 2016), it may be possible that women’s perception of financial hardship contributes to men’s awareness of the difficulties they face in fulfilling their social role, impacting their emotional well-being as a result.

We also found interesting associations between maternal and paternal reports about stressful events, depression and chaos perception, which suggest complex, bidirectional relationships within the parental couples’ perceptions. For instance, maternal reports of higher stressful events before the pandemic were related to fathers’ perceptions of home chaos later on during the pandemic. Similarly, paternal reports of more stressful events were associated with maternal depression and chaos perception during the pandemic. Stressed and/or depressed parents may be less likely to support their partner during the pandemic and to effectively manage the additional challenges related to childcare and domestic work, thus imposing a burden on their partner and possibly negatively affecting their emotional well-being.

When comparing parental reports from mothers and fathers, interesting differences were found. For instance, mothers reported higher overall levels of home chaos and depression than fathers before the beginning of the COVID-19 pandemic. Then during the pandemic mothers experienced more lockdown days than fathers. Previous studies have shown that women experience higher rates of depression than men, especially during their reproductive years. This phenomenon is associated with diverse neurobiological aspects (Eid et al., 2019), but also with the implication of women’s disproportionate engagement in caring activities compared to men. In Chile, mothers devote double the time that men do to caring for family members (Servicio Nacional de la Mujer, 2009; Comunidad Mujer, 2017), which may explain why mothers reported longer lockdown duration than their partners. Fathers still have low participation in co-parenting tasks. Data from 1,600 households in 2020 showed that 57% of fathers reported dedicating zero time to childcare activities for those under 14 years of age, which implies that women dedicate 3.2 more hours per day on average than men to unpaid domestic work (Encuesta de Bienestar Social, 2021).

The results of our moderation analyses suggest that both mothers’ and fathers’ self-criticism and dependency personality traits contributed to their depression symptoms, and those individuals with higher levels of self-criticism reported a larger increase in depressive symptomatology than parents with low levels of self-criticism. Self-criticism has not only been related to a higher physiological reactivity to stress (Silva et al., 2017), but also to deficiencies in the response to reward (favoring anhedonia) (Silva et al., 2018), which could explain this greater association with depression in our study. Hence, our findings support Blatt’s theory of depressive personality style, suggesting that both self-criticism and dependency traits predispose individuals to developing depression (Blatt, 2008). Accordingly, two recent systematic reviews (McIntyre et al., 2018; Werner et al., 2019) showed that self-criticism not only contributes to depression, but is also involved in the development of diverse mental health problems; thus, it can be viewed as a transdiagnostic process.

When exploring aspects associated with home chaos, we found that families with more children, who reported more stress events and parents with high self-criticism reported higher levels of home chaos at the beginning of pandemic lockdown than their counterparts. In this regard, self-criticism is associated with harsh standards, excessive striving for achievement, and a high need for acknowledgment (Blatt, 2008; Stoeber et al., 2008). Campos et al. (2018) also suggest that self-criticism is associated with less effective coping and adaptation strategies. It may be possible that parents who report high self-criticism traits have very high expectations of themselves and their children and may have difficulties adapting to and coping with family-related challenges. Parents with these characteristics may be reluctant to lower their high standards in terms of work performance, home organization, and children’s school performance, and may use positive and negative control strategies to restrict home chaos.

The changes in the perception of home chaos from the period before the pandemic to the lockdown was dependent on the levels of negative parental control. Surprisingly, families who reported the least increase in chaos from T1 to T2 were families with parents who displayed higher levels of negative control before the pandemic. This suggests that more restrictive parents were able to manage home disorganization better when the lockdown began. However, these effects disappeared by our third assessment (i.e., 40 days later), in line with studies showing that negative control increases short-term compliance in children, yet produces negative, long-term consequences for children’s socioemotional development (Berthelon et al., 2020). Future studies could examine possible associations between parental self-criticism, parenting practices, and child development.

The results of this study are limited by the sample size and characteristics (i.e., cohabiting families of children 3 to 4 years of age), so they cannot be generalized to families with single parents and/or older or younger children. Also, the assessment of mental health and home chaos was conducted using self-report measures. Nevertheless, the repeated-measures design allowed us to examine changes in these variables across the course of the COVID-19 pandemic in a sample of Chilean families which is representative of the middle-class population as explained in the description of the participants.

## Conclusion

Sanitary restrictions put in place to respond to the first pandemic outbreak had a strong impact on families’ mental health and organization. This study examines specific risk and protective factors that should be considered for adapting to non-normative social stressors, such as the COVID-19 pandemic. Our findings evidence how changes in parental self-reported depression and perception of home organization before and during the first month of strict lockdown in Chile were associated with financial stability level, personality traits, recently experienced major life events, and number of children. These results can offer relevant insight for health care professionals and public policy designers who must focalize resources and interventions for reducing the negative impact of the sanitary restrictions in light of vulnerability factors.

## Data Availability Statement

The raw data supporting the conclusions of this article will be made available by the authors, without undue reservation.

## Ethics Statement

The studies involving human participants were reviewed and approved by the Comité de Ética Institucional de Investigación-Universidad del Desarrollo. Written informed consent to participate in this study was provided by the participants and in case of minors by the participants or their legal guardian/next of kin.

## Author Contributions

JP participated in the analysis, development, and design of the article. DA participated in the article design and development. SC participated in the writing and development of the manuscript. AV-C participated in the data collection, development, and design of the article. EG participated in the article development. JS participated in the experimental design and overall review of the manuscript. All authors contributed to the article and approved the submitted version.

## Conflict of Interest

The authors declare that the research was conducted in the absence of any commercial or financial relationships that could be construed as a potential conflict of interest.

## Publisher’s Note

All claims expressed in this article are solely those of the authors and do not necessarily represent those of their affiliated organizations, or those of the publisher, the editors and the reviewers. Any product that may be evaluated in this article, or claim that may be made by its manufacturer, is not guaranteed or endorsed by the publisher.

## Supplementary Material

Supplementary Figure 1 Recruitment Flyer.

Supplementary Table 1 Checklist for Reporting Results of Internet E-Surveys (CHERRIES).
