# Supplementary figures and images for: Adapting to Adversity: Effects of COVID-19 on Parenting in Chile
Source: Front Psychol. 2022 Jul 5;13:868817. doi: 10.3389/fpsyg.2022.868817 (PMC9294446; doi:10.3389/fpsyg.2022.868817)

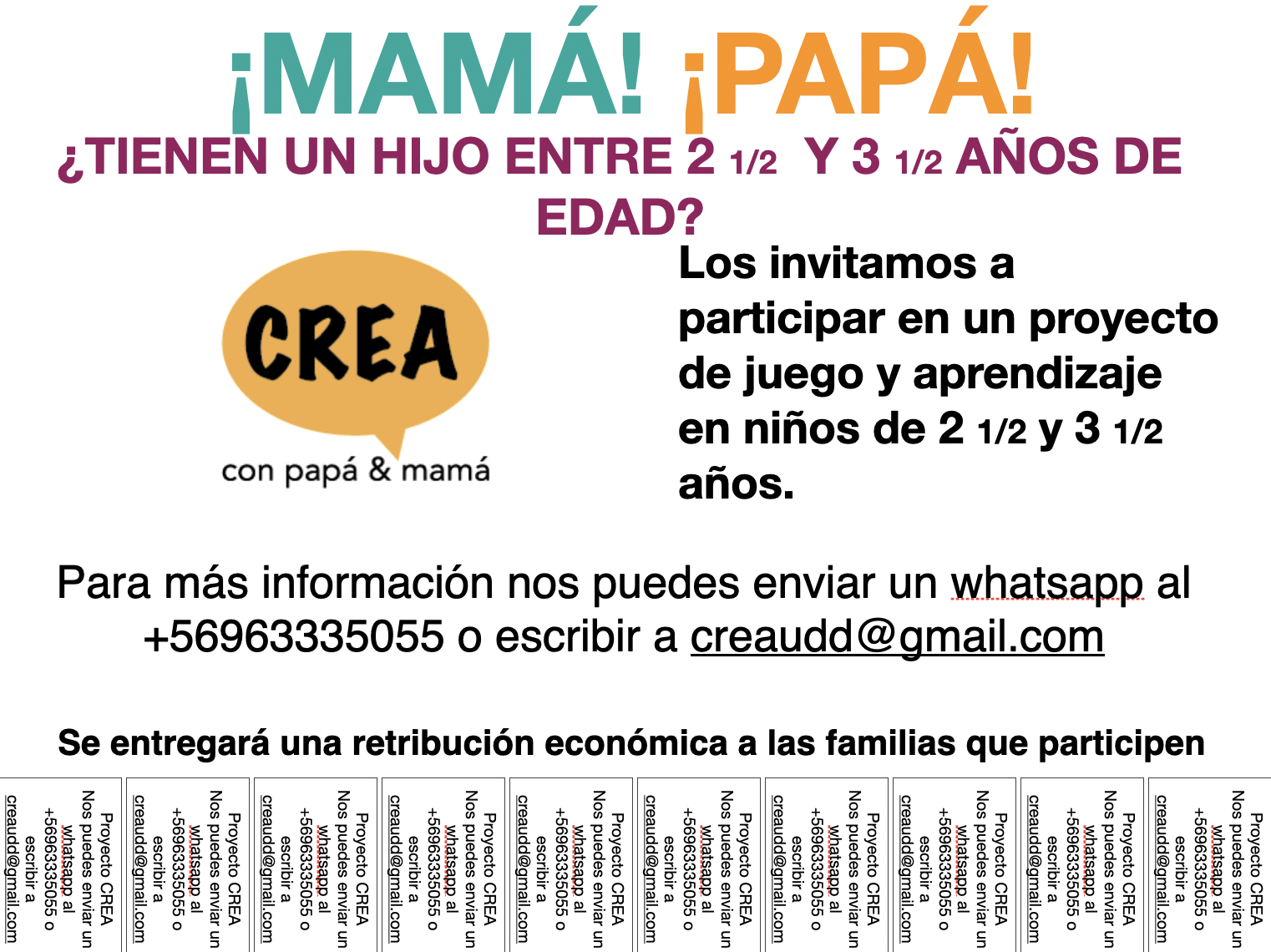

Supplement: Supplementary Figure 1 — Recruitment Flyer. [file Image_1.PNG]
