# Supplementary material for: Adapting to Adversity: Effects of COVID-19 on Parenting in Chile
Source: Front Psychol. 2022 Jul 5;13:868817. doi: 10.3389/fpsyg.2022.868817 (PMC9294446; doi:10.3389/fpsyg.2022.868817)
Supplement: Supplementary Table 1 — Checklist for Reporting Results of Internet E-Surveys (CHERRIES). [file Table_1.DOCX]

**Checklist for Reporting Results of Internet E-Surveys (CHERRIES)**

| ***Checklist Item*** | ***Explanation*** | ***Manuscript quote*** | ***Comments*** |
| --- | --- | --- | --- |
| Describe survey design | Describe target population, sample frame. Is the sample a convenience sample? (In “open” surveys this is most likely.) | This study used a repeated-measures design to follow up a sub-sample of 68 families who were involved in a larger study from the period before the onset of the Covid-19 pandemic (T1) to the beginning of the lockdown restrictions (T2) and 40 days later (T3). Inclusion criteria were: 1) being adult (i.e., 18 years of age or older), 2) cohabiting parents, 3) with a child aged 3-4 years at T1. Exclusion criteria for parents referred to diagnosis of an intellectual impairment, neurodevelopmental or severe psychiatric disorder (i.e., schizophrenia, bipolar disorder, major depressive disorder). | Nothing to add. |
| IRB approval | Mention whether the study has been approved by an IRB. | Study procedures were approved by the University Institutional Review Board. | Nothing to add. |
| Informed consent | Describe the informed consent process. Where were the participants told the length of time of the survey, which data were stored and where and for how long, who the investigator was, and the purpose of the study? | For T2 and T3 families were contacted via phone to invite them to participate in the follow-up study. Families who agreed and provided informed consent completed a set of online questionnaires when mandatory quarantine was applied in Santiago (T2) and 40 days later (T3). | New version: For T2 and T3 families were contacted via phone to invite them to participate in the follow-up study. Through this conversation participants were informed on the study characteristics and had the opportunity to ask questions about the procedure. Families received the study information sheet and informed consent which stated the study general purpose, instruments’ length and contact details of the principal investigator. Participants’ who agreed completed a set of online questionnaires when mandatory quarantine was applied in Santiago (T2) and 40 days later (T3). |
| Data protection | If any personal information was collected or stored, describe what mechanisms were used to protect unauthorized access. | No information | Any personal information about participants (e.g. as contact information) was destroyed after the data-collection process. |
| Development and testing | State how the survey was developed, including whether the usability and technical functionality of the electronic questionnaire had been tested before fielding the questionnaire. | No information | The selected questionnaires were uploaded to the survey’s platform and previously piloted to ensure correct functionality. |
| Open survey versus closed survey | An “open survey” is a survey open for each visitor of a site, while a closed survey is only open to a sample which the investigator knows (password-protected survey). | No information | Participants’ who agreed completed a set of online questionnaires with closed survey format, when mandatory quarantine was applied in Santiago (T2) and 40 days later (T3). |
| Contact mode | Indicate whether or not the initial contact with the potential participants was made on the Internet. (Investigators may also send out questionnaires by mail and allow for Web-based data entry.) | For T1, we recruited participants through wall-posters or by approaching them in the waiting rooms of four primary health care centers in the south area of Chile’s capital, Santiago. We contacted interested families by phone for further explanation of the project and scheduled an appointment for the assessment, performed at the health care center. Each family received financial compensation and children were given a sticker for their participation. For T2 and T3 families were contacted via phone to invite them to participate in the follow-up study. | Nothing to add |
| Advertising the survey | How/where was the survey announced or advertised? Some examples are offline media (newspapers), or online (mailing lists – If yes, which ones?) or banner ads (Where were these banner ads posted and what did they look like?). It is important to know the wording of the announcement as it will heavily influence who chooses to participate. Ideally the survey announcement should be published as an appendix. | For T1, we recruited participants through wall-posters or by approaching them in the waiting rooms of four primary health care centers in the south area of Chile’s capital, Santiago. We contacted interested families by phone for further explanation of the project and scheduled an appointment for the assessment, performed at the health care center. Each family received financial compensation and children were given a sticker for their participation. For T2 and T3 families were contacted via phone to invite them to participate in the follow-up study. | Recruitment announcement is published in the supplementary material. |
| Web/E-mail | State the type of e-survey (eg, one posted on a Web site, or one sent out through e-mail). If it is an e-mail survey, were the responses entered manually into a database, or was there an automatic method for capturing responses? | No information | Participants’ who agreed completed a set of online questionnaires through Survey Monkey platform with closed survey format. The questionnaires were answered when mandatory quarantine was applied in Santiago (T2) and 40 days later (T3). |
| Context | Describe the Web site (for mailing list/newsgroup) in which the survey was posted. What is the Web site about, who is visiting it, what are visitors normally looking for? Discuss to what degree the content of the Web site could pre-select the sample or influence the results. For example, a survey about vaccination on a anti-immunization Web site will have different results from a Web survey conducted on a government Web site | This does not apply to our study. | Nothing to add |
| Mandatory/ voluntary | Was it a mandatory survey to be filled in by every visitor who wanted to enter the Web site, or was it a voluntary survey? | This does not apply to our study | Nothing to add |
| Incentives | Were any incentives offered (eg, monetary, prizes, or non-monetary incentives such as an offer to provide the survey results)? | All participants received $7 USD at the end of their participation. | In T1 participants received an educational toy for their children. |
| Time/Date | In what timeframe were the data collected? | The questionnaires were answered when mandatory quarantine was applied in Santiago (T2) and 40 days later (T3). | For T1 questionnaires were answered by mothers and fathers on paper during the assessment in the health care center. |
| Randomization of items or questionnaires | To prevent biases items can be randomized or alternated. | No information | The order of the questionnaires were counterbalanced. |
| Adaptive questioning | Use adaptive questioning (certain items, or only conditionally displayed based on responses to other items) to reduce number and complexity of the questions. | This does not apply. | Nothing to add |
| Number of Items | What was the number of questionnaire items per page? The number of items is an important factor for the completion rate. | The instruments’ characteristics were described in detail or properly cited. | Nothing to add |
| Number of screens (pages) | Over how many pages was the questionnaire distributed? The number of items is an important factor for the completion rate. | No information. | Each questionnaire used one page on screen being in total 5 pages long. |
| Completeness check | It is technically possible to do consistency or completeness checks before the questionnaire is submitted. Was this done, and if “yes”, how (usually JAVAScript)? An alternative is to check for completeness after the questionnaire has been submitted (and highlight mandatory items). If this has been done, it should be reported. All items should provide a non-response option such as “not applicable” or “rather not say”, and selection of one response option should be enforced. | This does not apply to our study. | Participants had access to review their responses before submitting their answers. |
| Review step | State whether respondents were able to review and change their answers (eg, through a Back button or a Review step which displays a summary of the responses and asks the respondents if they are correct). | No information | Nothing to add |
| Unique site visitor | If you provide view rates or participation rates, you need to define how you determined a unique visitor. There are different techniques available, based on IP addresses or cookies or both. | This does not apply to our study | Nothing to add |
| View rate (Ratio of unique survey visitors/unique site visitors) | Requires counting unique visitors to the first page of the survey, divided by the number of unique site visitors (not page views!). It is not unusual to have view rates of less than 0.1 % if the survey is voluntary. | This does not apply to our study | Nothing to add |
| Participation rate (Ratio of unique visitors who agreed to participate/unique first survey page visitors) | Count the unique number of people who filled in the first survey page (or agreed to participate, for example by checking a checkbox), divided by visitors who visit the first page of the survey (or the informed consents page, if present). This can also be called “recruitment” rate. | No information | The recruitment process was done for 5 days at the end of April 2020. From the total sample of T1, 124 families were contacted by phone. One family left an email as their contact details. Each family was contacted by phone for up to three attempts. If there was no response or the phone number was no longer available the family was marked as “unreachable”. This was the case for 38% of the T1 sample. Of the 77 families that were invited to be part of T2 and T3, 78% agreed to participate. Therefore, 17 families decided not to be part of the present study and the final sample of 68 families. |
| Completion rate (Ratio of users who finished the survey/users who agreed to participate) | The number of people submitting the last questionnaire page, divided by the number of people who agreed to participate (or submitted the first survey page). This is only relevant if there is a separate “informed consent” page or if the survey goes over several pages. This is a measure for attrition. Note that “completion” can involve leaving questionnaire items blank. This is not a measure for how completely questionnaires were filled in. (If you need a measure for this, use the word “completeness rate”.) | No information | We don’t have access to this information. |
| Cookies used | Indicate whether cookies were used to assign a unique user identifier to each client computer. If so, mention the page on which the cookie was set and read, and how long the cookie was valid. Were duplicate entries avoided by preventing users access to the survey twice; or were duplicate database entries having the same user ID eliminated before analysis? In the latter case, which entries were kept for analysis (eg, the first entry or the most recent)? | This does not apply to our study | Nothing to add |
| IP check | Indicate whether the IP address of the client computer was used to identify potential duplicate entries from the same user. If so, mention the period of time for which no two entries from the same IP address were allowed (eg, 24 hours). Were duplicate entries avoided by preventing users with the same IP address access to the survey twice; or were duplicate database entries having the same IP address within a given period of time eliminated before analysis? If the latter, which entries were kept for analysis (eg, the first entry or the most recent)? | This does not apply to our study | Nothing to add |
| Log file analysis | Indicate whether other techniques to analyze the log file for identification of multiple entries were used. If so, please describe. | No information | Participants’ ID was assigned in the survey’s link. This allowed us to identify duplicated or wrong entries. |
| Registration | In “closed” (non-open) surveys, users need to login first and it is easier to prevent duplicate entries from the same user. Describe how this was done. For example, was the survey never displayed a second time once the user had filled it in, or was the username stored together with the survey results and later eliminated? If the latter, which entries were kept for analysis (eg, the first entry or the most recent)? | No information | Duplicated entries from participants were deleted. The most completed responses were used for data analysis. |
| Handling of incomplete questionnaires | Were only completed questionnaires analyzed? Were questionnaires which terminated early (where, for example, users did not go through all questionnaire pages) also analyzed? | No information | Incomplete questionnaires or participant’s with missing items were included in the analysis. |
| Questionnaires submitted with an atypical timestamp | Some investigators may measure the time people needed to fill in a questionnaire and exclude questionnaires that were submitted too soon. Specify the timeframe that was used as a cut-off point, and describe how this point was determined. | This does not apply to our study. | Nothing to add |
| Statistical correction | Indicate whether any methods such as weighting of items or propensity scores have been used to adjust for the non-representative sample; if so, please describe the methods. | This does not apply to our study. | Nothing to add |

This checklist has been modified from Eysenbach G. Improving the quality of Web surveys: the Checklist for Reporting Results of Internet E-Surveys (CHERRIES). J Med Internet Res. 2004 Sep 29;6(3):e34 [erratum in J Med Internet Res. 2012; 14(1): e8.]. Article available at [https://www.jmir.org/2004/3/e34](https://www.jmir.org/2004/3/e34/)/; erratum available <https://www.jmir.org/2012/1/e8/>. Copyright ©Gunther Eysenbach. Originally published in the [Journal of Medical Internet](http://www.jmir.org) Research, 29.9.2004 and 04.01.2012.

This is an open-access article distributed under the terms of the Creative Commons Attribution License (<https://creativecommons.org/licenses/by/2.0/>), which permits unrestricted use, distribution, and reproduction in any medium, provided the original work, first published in the Journal of Medical Internet Research, is properly cited.
